# Assessing and Forecasting ISR-Affected Critical Infrastructure: State-of-the-Art and Challenges

**DOI:** 10.3390/ma17010188

**Published:** 2023-12-29

**Authors:** Ana Bergmann, Rennan Medeiros, Leandro Sanchez

**Affiliations:** Department of Civil Engineering, Faculty of Engineering, University of Ottawa, Ottawa, ON K1N 6N5, Canada; rdasi077@uottawa.ca (R.M.); leandro.sanchez@uottawa.ca (L.S.)

**Keywords:** internal swelling reactions, diagnosis and prognosis, alkali–aggregate reaction, delayed ettringite formation

## Abstract

Internal swelling reactions (ISRs) are among the most critical deterioration mechanisms affecting infrastructure’s durability worldwide. While preventative measures for new structures have been extensively explored, effective protocols for diagnosing and prognosing ISR-affected structures, especially at their early stages, are still required. Therefore, through a comprehensive bibliometric analysis, this study focuses on exploring the evolution and current methods for assessing and forecasting ISR damage in concrete structures. For diagnosis, a shift from concrete petrography and non-destructive techniques (NDTs) towards more comprehensive methods (i.e., multi-level assessment) with the stiffness damage test (SDT) and damage rating index (DRI) is observed. Moreover, it identifies the valuable inputs from residual expansion and pore solution analysis as relevant parameters for prognosis. Based on these findings, a structured management framework is proposed aiming to refine the diagnosis and prognosis processes of ISR-affected infrastructure, ultimately contributing to improved long-term structural health and maintenance strategies.

## 1. Introduction

Internal swelling reactions (ISRs) critically affect the durability, serviceability, and overall performance of concrete infrastructure. These reactions, which primarily include alkali–aggregate reaction (AAR), internal sulfate attacks (i.e., notably DEF), and freeze and thaw (FT) cycles, tend to occur in the presence of moisture, leading to concrete expansion and deterioration, resulting in reduced mechanical properties [1,2,3,4].

Efforts to prevent ISR in new concrete structures have led to significant research on developing laboratory tests to assess the potential of its occurrence [5,6,7]. Simultaneously, the effectiveness of different material combinations (i.e., cement type, supplementary cementitious materials) has been explored to mitigate ISR mechanisms and improve field performance [8,9,10,11,12]. Despite the advances in preventative measures for new structures, concerns remain about effectively diagnosing, prognosing, and maintaining existing ISR-affected structures.

Current diagnostic protocols are limited to petrographic examination, superficial crack evaluation, and chemical analysis to identify the cause(s) of damage, while quantifying its extent remains primarily qualitative [13,14,15]. Regarding prognosis, these protocols rely on residual expansion and soluble alkalis, yet these indicators have not been fully explored. Furthermore, maintenance strategies typically include finite element analysis for risk assessment but often lack detailed repair and rehabilitation strategies.

In this context, the development of a reliable management framework integrating diagnosis (i.e., damage cause and extent) and prognosis (i.e., potential for further distress) is essential to guide the selection of rehabilitation strategies and intervention schedules. However, due to the unique nature of each ISR mechanism, developing such a comprehensive management framework may be challenging. It requires careful consideration of different factors, such as aggregate reactivity, structural geometry, environmental conditions, reinforcement configuration, and the effects of possible coupled deterioration mechanisms.

Therefore, aiming to understand the currently employed laboratory and field methods for ISR assessment and forecasting, this paper performs an extensive bibliometric analysis. The final goal is to propose a structured framework outlining the main techniques for ISR evaluation, emphasizing their practical application for accurate diagnosis and prognosis. The expected outcome is to enhance strategies for the maintenance of critical infrastructure affected by ISR, extending their service life, and ensuring sustained safety.

## 2. Internal Swelling Reactions (ISRs)

Internal swelling reactions (ISRs) are deterioration processes that can lead to concrete expansion and deterioration, usually in the presence of moisture. ISR can reduce mechanical properties, compromising the long-term performance of affected concrete. Among ISR mechanisms, the primary ones are alkali–aggregate reaction (AAR), internal sulfate attacks (i.e., DEF), and freeze and thaw (FT) cycles [1,2,3,4].

### 2.1. Alkali–Aggregate Reaction (AAR)

Alkali–aggregate reaction (AAR) is a chemical reaction between the alkalis in the concrete matrix and reactive minerals in the aggregates [1]. This process leads to the formation of microcracking and, consequently, a reduction in the mechanical performance and decreased durability of concrete structures. The rate of AAR development is affected by the type of reactive minerals in the aggregates, the content of alkali hydroxides, and environmental conditions (i.e., moisture and humidity) [7,16,17,18]. AAR comprises two distinct types: the alkali–silica reaction (ASR) and the alkali–carbonate reaction (ACR).

**Figure 1:**
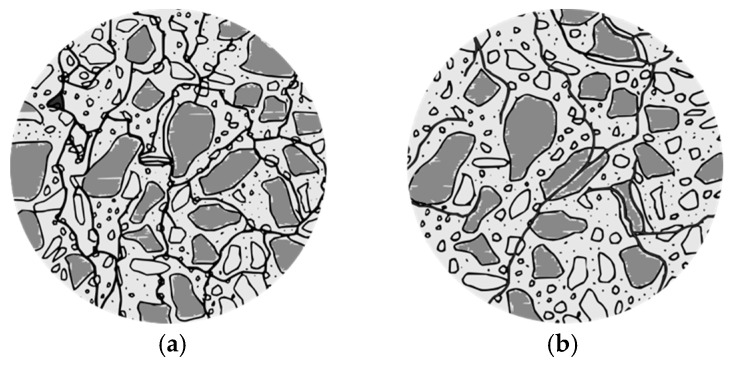
Alkali–aggregate reaction cracking pattern: (**a**) ASR from the reactive sand; (**b**) ASR from the reactive coarse aggregate. Adapted from [22].

(a) Alkali–carbonate reaction (ACR) is the reaction between carbonate rocks, such as dolomitic limestone, and alkali hydroxides from concrete pore solution. ACR results in cracks most prominently located near cement paste and adjacent aggregates, without secondary products being formed [1,19]. ACR-related damage can become apparent in concrete structures within a relatively short period of time (i.e., five years).
(b) Alkali–silica reaction (ASR), the more frequent form of AAR, occurs primarily in aggregates containing reactive siliceous materials like poorly crystallized siliceous minerals, volcanic glass, or quartz-bearing rocks [20,21]. In ASR, hydroxyl ions from the cement paste attack these siliceous aggregates, leading to the formation of a secondary product (i.e., ASR gel). This gel can absorb water and expand, causing internal pressure and cracking throughout the aggregate and cement paste (Figure 1). The mechanical property losses due to ASR are dependent on the extent of concrete expansion [22]. With minor expansions (0.0–0.05%), reductions in modulus of elasticity and tensile strength are noticeable, ranging up to 30% and 30–70%, respectively, while compressive strength experiences a minimal reduction of about 5%. Moderate expansion (0.12%) exacerbates the cracking in aggregates and reaches the cement paste, with the rate of decrease in tensile strength and modulus of elasticity slowing down and compressive strength diminishing by 10%. Higher expansions (0.20%) extend the cracking into the cement paste, with a 25% reduction in compressive strength. At an expansion rate of 0.30%, a network of interconnected cracks forms, potentially leading to a 40% loss in compressive strength.

### 2.2. Internal Sulphate Attack (ISA)

#### 2.2.1. Delayed Ettringite Formation (DEF)

Delayed ettringite formation (DEF) is a type of sulfate attack occurring after concrete setting [23,24]. This mechanism is triggered when the concrete setting temperature exceeds 65–70 °C, leading to the initial formation of poorly crystalline monosulfate. As the temperature drops and moisture penetrates, sulfate and alumina released from the calcium silicate hydrate (CSH) promote the conversion of monosulfate into ettringite. This conversion, occurring in microscopic pores, may cause the cement paste to expand and the concrete to crack.

**Figure 2:**
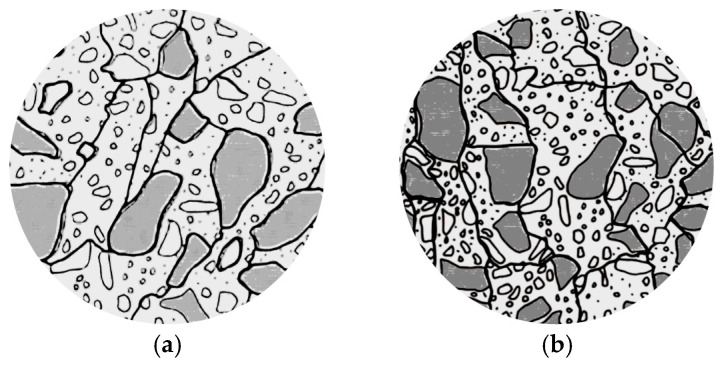
Cracking pattern: (**a**) Delayed ettringite formation—DEF; (**b**) Freeze and thawing—FT. Adapted from [22].

Concrete damaged by DEF loses mechanical properties due to cracking in the interfacial transition zone (ITZ). With moderate expansions (0.12%), the concrete’s modulus of elasticity and compressive strength losses are in the range of 50% and 10%, respectively. With greater expansions (between 0.20% and 0.30%), existing cracks continue to connect and form a network in the concrete paste (Figure 2a), while some new cracks appear in the aggregate. The network becomes stronger and denser when its expansion exceeds 0.30%. Over 0.50% of expansion, aggregate particles start to disaggregate. Hence, DEF-damaged concrete can lose 85% of its modulus of elasticity and 50% of its comprehensive strength [22].

Additionally, DEF can co-occur with ASR, as the mechanism promotes ion mobility within the concrete, facilitating DEF. The presence of alkalis is a requisite for ettringite stability, linking the mechanisms of ASR and DEF.

#### 2.2.2. Sulfide Bearing Aggregates

A range of mineral sulfides, including pyrite (Fe_2_S) and pyrrhotite (Fe_1−x_S), are commonly present in aggregates. When these sulfides are exposed to water and oxygen, they oxidize, resulting in the generation of sulfuric acid [25]. As a result, the sulfuric acid reacts with the components of cement paste, leading to an internal sulfate attack characterized by the formation of gypsum, ettringite, thaumasite, and iron oxides, commonly referred to as “rust” [26].

The likelihood of such minerals causing deterioration in concrete is influenced by several factors, including chemical composition, crystal structure, surface area, and the extent of their exposure. The alkaline environment typical of concrete further enhances sulfide oxidation, making sulfide-bearing aggregates particularly aggressive agents of internal sulfate attack [27].

### 2.3. Freeze and Thawing (FT)

Freeze–thaw deterioration in concrete is driven by the expansion of water as it freezes within the capillary pores, a process highly dependent on environmental conditions. The source of moisture can be intrinsic (i.e., the pores of concrete) or extrinsic (i.e., rain). The critical issue arises when the spatial arrangement of the voids within the concrete does not allow for the free movement of water to the air voids, leading to internal stresses during water freezing.

To mitigate this, the spacing factor should be optimized, typically between 200 µm and 250 µm, by adjusting the air content in the concrete mix. This adjustment ensures that there is enough space in the air voids to accommodate the expansion of water upon freezing without extra pressure on the cement paste [28].

In terms of damage features, FT manifests as cracks within the cement paste, the interfacial transition zone (ITZ), and aggregates (Figure 2b). These defects can significantly compromise the concrete’s structural integrity, reducing the modulus of elasticity by 35% and compressive strength by 30% with only low expansion. At expansions over 0.12%, cracks increase within the cement paste, and beyond 0.30% expansion, an interconnected network of cracks forms, further affecting the modulus of elasticity and compressive strength by as much as 50% and 40%, respectively [22].

## 3. Materials and Methods

The bibliometric analysis employed in this study serves as a systematic review of the predominant techniques in the literature for assessing and forecasting ISR in critical infrastructure. This analysis, based on a keyword frequency method, resulted in a comprehensive understanding of the available research. While this approach might lean towards more frequently cited publications, the following procedure was adopted, aiming to reduce potential biases and reflect relevant studies in the field.

### 3.1. Keyword Identification and Database Search

The initial phase involved identifying a set of keywords central to the research topic. To ensure a comprehensive search strategy, these keywords were carefully chosen based on their relevance to ISR, aiming to incorporate scientific programs on ISR-affected existing structures. The use of Boolean logic (i.e., “AND,” “OR,” “NOT”) combined with the keywords aimed to narrow and adjust the research topics. For instance, “AND” is incorporated to include publications with specific terms, while the “OR” approach expands the search to include the suggested terms. The following keyword string was used for the database search in the Web of Science:

- ((“INTERNAL” AND “SWELLING” AND “REACTIONS”) OR (“DELAYED” AND “ETTRINGITE”) OR (“ALKALI” AND “SILICA” AND “REACTION”)) AND (“PROGNOSIS” OR “DIAGNOSIS” OR “MANAGEMENT” OR “ASSESSMENT” OR “FORECAST” OR “ASSESSING” OR “FORECASTING”)

### 3.2. Category Selection and Validation

To ensure the relevancy of the results, the search was refined to the specific categories pertinent to civil engineering available in the database platform (i.e., Web of Science). These categories included Construction Building Technology, Materials Science Multidisciplinary, and Engineering Civil, among others, representing the target publications.

### 3.3. Data Extraction and Analysis

From the refined search, 465 publications were identified, indicating correspondence to the topic. The subsequent phase involved text analysis and cataloging the frequency of occurrences of keywords. To represent the most relevant terms and visually represent the net, only keywords with at least three occurrences were included in the net analysis, resulting in 245 keywords meeting this criterion.

### 3.4. Final Selection and Terms for Analysis

Among the 245 keywords, the final selection phase concentrated on isolating terms directly related to ISR assessment. A total of 57 terms were selected, such as “accelerated test,“ “damage rating index (DRI),“ “elastic-wave spectroscopy,“ and “X-ray diffraction (XRD).“ These terms represented a wide range of techniques and concepts relevant to ISR assessment, from experimental methods (i.e., residual expansion) to analytical approaches (i.e., finite element modeling).

### 3.5. Bibliometric Tool Usage

For the evaluation of the identified keywords, VOSviewer v1.6.19 software was employed. This tool facilitated the visualization of keyword trends and their interconnections over time, providing an insightful overview of the evolution and scope of research in the field of ISR assessment of critical infrastructures.

## 4. Bibliometric Analysis and Trends in ISR Assessment

**Figure 3:**
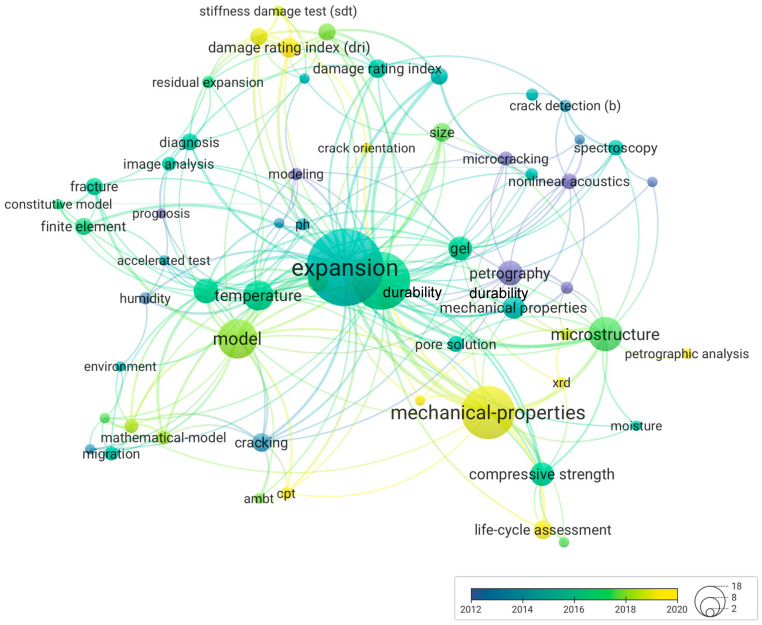
Bibliometric analysis of keywords used in published articles. Available at http://tinyurl.com/2agxmzd7, 19 November 2023.

Figure 3 illustrates the results obtained from the bibliometric analysis, showcasing the most frequently used keywords across 465 publications and highlighting the central themes associated with ISR assessment within concrete structures. The network graph shows strong interconnection among “expansion” and “model,” as well as “durability” and “mechanical properties,” indicating their significant roles in the ISR context.

Additionally, Figure 3 traces the temporal evolution of trends in assessing ISR-damaged structures. Initially, around 2012, the investigation relied on “petrography” and “microcracking” to understand ISR manifestations. This focus expanded to include “non-destructive techniques” (NDTs), such as nonlinear acoustics and spectroscopy, reflecting the progression of the topic.

In the subsequent period (2014–2016), there was a notable inclusion of “image analysis” alongside the implementations of multi-level assessment techniques, including the “stiffness damage test” (SDT) and “damage rating index” (DRI). Besides the diagnosis, “finite element modeling” and “residual expansion tests” became apparent in 2016–2018, indicating a first move toward the future behavior of affected structures. This trend was further enhanced as indicated by a strong connection between “concrete microstructure” and “mathematical models,” suggesting a more analytical approach to ISR understanding and forecasting.

**Figure 4:**
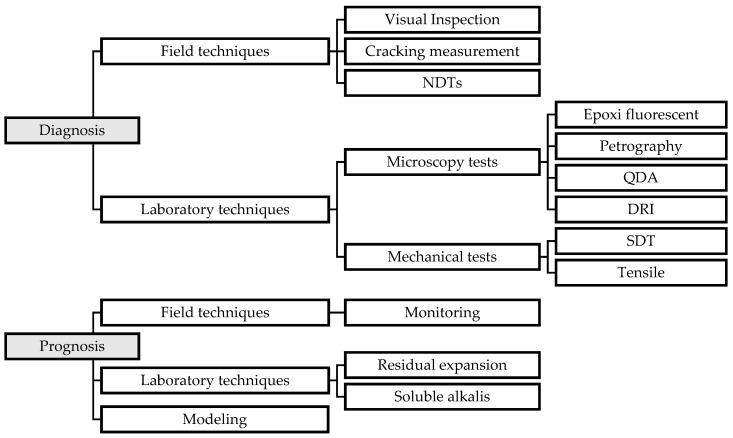
ISR-affected concrete diagnosis and prognosis are explored in this study.

By summarizing the bibliometric analysis, Figure 4 categorizes the observed methodologies into diagnosis and prognosis phases, providing a structured overview of the main methods employed in the assessment of ISR-affected structures. The diagnosis includes field techniques such as visual inspection, cracking measurement, and NDTs. This extends to laboratory analysis involving microscopic (i.e., epoxy fluorescence, petrography, quantitative damage analysis—QDA, DRI) and mechanical tests (i.e., SDT and tensile). For prognosis, the categories are divided into field techniques (i.e., monitoring), laboratory techniques (i.e., residual expansion and soluble alkalis), and modeling.

## 5. Diagnosis of ISR-Damaged Concrete

### 5.1. Field Techniques

Field assessment typically involves visual inspections and qualitative appraisals of the damage signs. In addition, crack measurements and non-destructive measures have been used to strengthen the preliminary ISR-distress structure diagnosis, supporting further tests [29,30,31,32,33,34,35,36,37,38].

#### 5.1.1. Visual Qualitative Symptoms

Visual inspection, one of the first steps in evaluating concrete infrastructure, identifies concrete’s condition at some point in its lifespan [39]. During this step, damages may only be observed subjectively and not quantitatively assessed in a structure. Therefore, such a technique can have varying results based on the professional’s level of experience and the damage’s extent.

For instance, the external distress symptoms of structures affected by ISR may be similar to those of other mechanisms, yet specific details support the preliminary qualitative assessment of the damage. During low damage degrees, ASR or DEF may produce microcracking with expansive-related products, but external inspection reveals no pattern. Consequently, diagnosing ASR and DEF based on qualitative visual symptoms is challenging when the damage degree is low.

In contrast, if the degree of damage is medium to high, it is easier to observe some traces of evidence, such as alterations to joint closure (pavements, Jersey barriers, and dam), structural misalignments, ISR external cracking patterns (i.e., crack maps or alignment with the confinement), discoloration of surfaces near cracks, and exudation of white or yellowish products around cracks. Yet, when the damage reaches high degrees, the typical signs are more pronounced and easily identified throughout the damaged structural members [13,14,40].

#### 5.1.2. Cracking Measurements

Analyzing the patterns and extent of cracking on concrete surfaces can provide valuable insights into the internal behavior and the extent of damage within the concrete structure. To facilitate this, measuring techniques have been developed and utilized for monitoring and assessing concrete cracking. The following subsections will detail the most commonly employed methods.

(a) Expansion index to date, established by the Institution of Structural Engineers [38].

**Figure 5:**
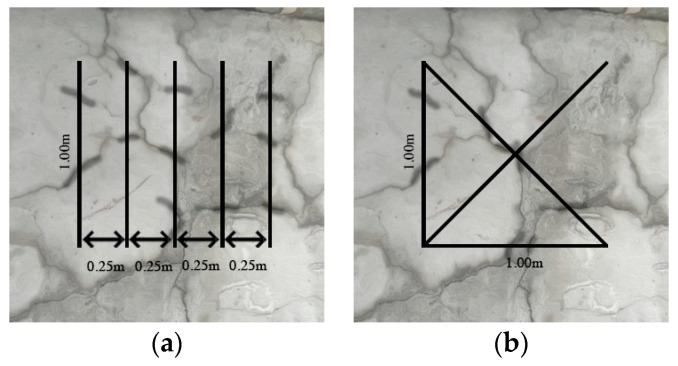
(**a**) Expansion index to date (**b**) Cracking index (CI) visual representation.

As shown in Figure 5a, this method involves a systematic approach to measuring cracks. It requires summing the widths of all cracks that intersect five straight lines, which are one meter in length each and spaced 0.25 m apart. After measuring, the severity of the damage is then categorized into one of five distinct levels, providing a structured assessment of the crack impact.

(b) LCPC-cracking index proposed by In this case, the Laboratoire Central Ponts et Chaussées (LCPC) [37].

As shown in Figure 5b, this method involves measuring the crack widths and lengths. For this, 1 m × 1 m squares are marked on the concrete surface, with each square having a horizontal bottom line, a vertical left line, and an X-line connecting the edges of the frame. Only the cracks that intersect these lines are considered for measurement. The cracking index (CI) is then calculated by dividing the total width of these cracks by their cumulative length, expressed in millimeters per meter (mm/m). The resulting CI is categorized into levels ranging from negligible (0–0.5) to ultra-high (>10), offering a gradation of crack severity.

#### 5.1.3. Non-Destructive Testing (NDT)

Among the numerous NDT techniques available for assessing concrete integrity, electrical resistivity, thermography, ground penetrating radar (GPR), and acoustic methods have been employed to evaluate ISR-distressed concrete in the field [33,41,42].

(a) Electrical resistivity (ER)

ER has been used to assess ISR-distressed concrete [43] due to the swelling products possibly altering the concrete’s electrical properties. However, it is nevertheless possible to identify some variations when comparing the electrical resistivity with the extent of damage since the electrical properties of concrete are highly dependent on the concrete humidity [44].

(b) Surface thermography

Thermography is a qualitative method for detecting defects in surfaces (such as honeycombs or delamination) by measuring the temperature at the surface. A difference in surface temperature may improve the detection of ISR defects that are not readily apparent at first glance due to flaws in concrete that affect its heat conduction properties [45]. However, this methodology may not be suitable for detecting damage in depth.

(c) Ground penetrating radar (GPR)

GPR is an effective method of assessing concrete that has been damaged by ISR, enabling the classification of zones where ISR is affecting a structural member. GPR’s ability to assess distress in concrete caused by ISR is confirmed by a higher dielectric constant that identifies both ISR-affected and high moisture-content regions [43,46].

(d) Stress waves

Stress waves, generated primarily by acoustic methods, have been used to evaluate concrete structures affected by ISR. Linear acoustics (LAs) and nonlinear acoustics (NLAs) approaches have produced significant results. It has been demonstrated that LA techniques like ultrasonic pulse velocity (UPV) and linear wave attenuation are sensitive to low damage degrees. In such techniques, ultrasonic pulses travel over a known path, allowing the relative uniformity of concrete to be determined based on the measured velocity [46]. Meanwhile, using resonant frequency, frequency modulation, and harmonic generation, NLA methods have been used to assess ISR-distressed concrete with low, moderate, and high damage degrees with limited variation [44,47].

Although these techniques have been used to support the diagnosis of ISR-affected concrete, their accuracy and precision have proven to be higher in monitoring the escalation of ISR distress over time. Furthermore, these outcomes can be combined with sensors measuring displacement, humidity, and temperature and significantly improve ISR-damaged structures monitoring, such as fiber optic (local and distributed), vibrant wires, and strain gauges to measure strain; thermocouples to quantify temperature variation; and magnetic microwire embedded in cement-based composite (MMCC) to assess the stress/strain sensing properties of a cement-based material [48,49,50].

### 5.2. Laboratory Techniques

Once the results from the visual inspection confirm signs of distress and deterioration, laboratory techniques are employed for a more thorough assessment of the structure. The second phase involves microscopic analysis, mechanical testing, chemical analysis, ultrasonic imaging, and other specialized tests to determine the structure’s current condition.

#### 5.2.1. Microscopic Techniques

(a) Petrography (thin section microscopy)

Petrography studies rocks and their chemical, physical, structural, and mineralogic composition. In addition, it provides systematic classifications and precise descriptions of rocks and cementitious materials (i.e., concrete). To assess possible causes of deterioration in concrete, the petrographic analysis used is the thin section, one of the most widely used approaches. A polarizing petrographic microscope is used to examine concrete sections that have been impregnated with epoxy and have a thickness between 20 and 30 μm and a surface area of 75 mm × 50 mm (limited by the microscope’s stage capacity). The results of this microscopic analysis include the concrete’s composition, texture, microstructure, and physicochemical properties. Different methods, materials, epoxy types, and lights can be used to enhance and create contrast in the observed feature. An ASR investigation involves coating a finished concrete thin section with uranyl acetate and rinsing it after three minutes with water; then, the ASR gel absorbs the acetate. In this case, ASR gel fluoresces when analyzed under ultraviolet (UV) light, providing more accurate results and highlighting distress features. Depending on the case, there are a variety of coating materials that fluoresce under UV light [51].

(b) Epoxy fluorescent coated sections

The fluorescent epoxy coating is used to detect microcracks and distress within concrete structures. In this technique, the area of interest can be seen through a microscope by tagging the concrete section with a fluorescent epoxy material, allowing the cracks or other signs of distress to be captured with higher precision than other techniques, such as X-rays and ultrasound. Due to its higher accuracy, the method can also provide accurate pictures of damaged areas and the material phase where the distress is located (i.e., aggregate, cement paste, interfacial transition zone—ITZ). The epoxy fluorescent-coated samples’ outcome can be used to assess the cause and determine how the structure will perform under certain loads. This information allows future maintenance monitoring plans to be developed through informed decision-making [51].

(c) Damage Rating Index (DRI)

DRI is a semi-quantitative analysis approach used to assess the concrete’s damage degree, inner quality, and physical integrity. Even so, it distinguishes crack types, which contribute to determining the cause of distress when correlated to mechanical property losses; it requires long training hours to reduce its subjectivity factor. Considerable research has been performed over the years to adapt the DRI test, especially regarding its weighting factors [19,22,52,53]. Stereomicroscopy (15–16× magnification) is used in DRI for concrete samples polished with usually 200 cm^2^ areas extracted from affected structures and normalized to 100 cm^2^ areas. The damage features (e.g., cracks) are counted on the samples’ surface using 1 cm^2^ grids, and the number of counts, multiplied by weighing factors, determines the DRI number. The number of counts in DRI corresponds to each type of petrographic feature: cracks in the coarse aggregate, opened cracks in coarse aggregates, cracks with reaction product in the coarse aggregate, coarse aggregated debonded, cracks in cement paste, cracks with reaction product in cement paste, an air void lined/filled with gel, a reaction rim around the aggregate, disaggregate/corroded aggregate particles. As a final step, each type of petrographic feature is multiplied by weighting factors, as presented in Table 1, in order to balance its relative importance concerning the mechanism. Therefore, DRI value and petrographic features can be used to classify damage causes, and the correlation with other mechanical properties and losses can also be used to estimate the damage degree.

(d) Qualitative Damage Assessment (QDA)

QDA is performed in thin sections using a polarized microscope under 4× magnification, allowing the identification of cracks and the definition of cracking patterns. In QDA, deterioration features are rated from 1 to 5 according to the five deterioration classes determined by the damage rating index (DRI): negligible, negligible, marginal, moderate, high, and very high. The main deterioration features observed are cracks in the cement paste, cracks crossing coarse aggregate particles, debonded aggregate particles, cracks crossing the coarser fraction of fine aggregates, and the presence of ASR product in cracks (located in the aggregate or cement paste in pores, or at interfaces). Since the QDA is a qualitative test, it does not consider cracked cement paste, and cracks are considered artifacts during thin section preparation. Also, voids with ASR products without associated cracks are not considered to be signs of ASR. Open cracks in cement paste containing ettringite crystals are considered cracks in the cement paste rather than cracks with ISR products. Each thin section is divided into five regions, and the QDA class is determined by summing the frequency of the listed features [54].

#### 5.2.2. Mechanical Test

(a) Stiffness Damage Testing (SDT)

SDT can be used to quantify the degree of internal deterioration of damaged concrete, thereby providing references for mechanical property losses. For this method, the damage is understood as a reduction in mechanical properties (compressive and tensile strengths), stiffness (elastic modulus), and physical integrity/durability (intern cracking extent). Consequently, over the years, some contributions have been made to improve SDT results [55,56,57]. Currently, SDT is conducted by applying five loading/unloading cycles at a controlled loading rate of 0.10 MPa/s, using 40% of the sound concrete strength (f’c). The main parameters for determining the extent of damage in a specimen based on SDT outcomes are listed below.

**Figure 6:**
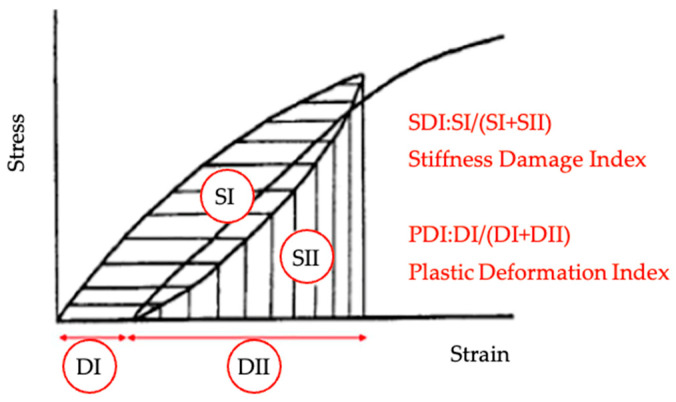
Fragility index (SII/SI) [22].

- Modulus of elasticity (ME, in GPa), which is related to the material’s stiffness and is measured by the secant slope average between the second and third cycles of the stress–strain curve (origin to 40% load);
- Hysteresis area (H, in J/m^3^), which shows the energy loss difference among damaged and undamaged samples, being the average between the four last cycles;
- The nonlinearity index (NLI, dimensionless) is proposed as the ratio of the slope of the stress–strain curve at half the maximum load over the secant modulus of elasticity. By analyzing the shape of the curve (concave or convex), the output is related to the crack’s orientation and damage degree. The higher the damage degree, the higher the difference between the slopes at the half and peak loads;
- The stiffness damage index (SDI, dimensionless) is calculated by dividing the dissipated energy (SI) by the total energy (SI + SII), as shown in Figure 4, from the loading and unloading cycles. The SDI measures the dissipated energy for crack closures over the test, and the first hysteresis area is usually greater than the rest, indicating most cracks have closed by this stage;
- The plastic deformation index (PDI, dimensionless) corresponds to the specimen plastic deformation (DI) divided by the total deformation of the system (DI + DII), as observed in Figure 6. It occurs when concrete cannot be returned to its original condition when unloaded due to crack surfaces becoming rough and even sliding across one another, resulting in unrecoverable strains.

Finally, a few parameters may influence SDT outputs, including moisture content (drier samples have less hysteresis area, less plastic deformation, and higher stiffness for the same damage degree); sample geometry (i.e., the length to the diameter should be equal to two); and exposure conditions, sample location, and direction (the more exposed the sample, the worse the damage).

(b) Tensile Strength

There has been evidence that tensile strength is much more affected by ISR than compressive strength, suggesting that such properties should be evaluated when assessing damaged concrete [22]. A few methods are available for assessing concrete’s tensile strength, including tensile splitting tests and gas pressure testing [58,59,60].

- Tensile splitting tests determine the splitting tensile strength of cylindrical concrete specimens. As a result of applying a diametrical compressive force along the length of a cylindrical specimen, tensile failure occurs since the load application area is under triaxial compression. Finally, the splitting tensile strength is calculated by dividing the maximum load by the parameters of the sample geometry [60].
- The gas pressure method developed by The Building Research Council of Waterford is reliable for evaluating tensile strength in deteriorated concrete samples. In this methodology, concrete test cylinders or cores are subjected to uniform pressure on the curved surface of the sample within a sealed cylindrical test chamber by compressed gas. Then, a monotonic increase in gas pressure is applied to the test cylinder until it fails in a plane transverse to its axis of rotation. It results from a hydrostatic reaction in the pore water and a biaxial reaction in the solid phase, culminating in a net internal tensile force generated from the pore fluid [22]. Therefore, this method is well suited for understanding the microstructure and integrity aspects of the cementitious matrix [58].

Table 2 summarizes the diagnostic methods for concrete structures affected by ISR; the main outcomes, sample specifications, and references can be observed.

## 6. Prognosis of ISR-Damaged Concrete

The prognosis of ISR-affected infrastructure must consider predicting future deterioration and the potential for future structural implications. The inputs for the prognosis usually come from field monitoring and laboratory tests, as well as mathematical modeling, which may be roughly divided into material (i.e., micromodels, micro-mesomodels, mesomodels) and structural (i.e., macroscale) scales.

### 6.1. Field Techniques

#### Monitoring strategies

Structural monitoring, essential for assessing the condition and performance of structures over time, implements tools and techniques to measure key parameters like displacement, strain, and temperature. These measurements are crucial for the early detection of potential issues, thereby preventing severe damage. Structural monitoring is generally of two types [61,62,63]:

(a) Structural performance monitoring (SPM): This evaluates the structure’s capacity based on analytical or numerical models over a specific period.
(b) Structural Health Monitoring (SHM): This involves continuous monitoring using sensors to capture real-time data, allowing for the detection and tracking of structural damage.

Early sensor installation allows for regular inspections without the need for invasive methods. This technology is interesting for both existing structures requiring damage monitoring and new construction in the sense of an ongoing assessment.

The key to understanding both the current state and future behavior of a structure lies in the data gathered over time. This includes insights into deterioration rates, reductions in loading capacity, water ingress, and temperature variations. The outcomes depend on the types of sensors used. Selection criteria for monitoring strategies should consider parameters to be measured, testing methods, frequency, number of test locations, and sensor placement considerations [61].

### 6.2. Laboratory Techniques

#### 6.2.1. Residual Expansion

One of the most important prognosis inputs is the ultimate or remaining expansion capacity of the ISR-affected concrete and the curve shape (i.e., indicating the swelling rate), as summarized in Table 3. A laboratory test to assess residual expansion is typically conducted on cores extracted from the distressed structure. In fact, samples are kept under controlled conditions in harsh environments to enhance ISR expansion. The expansion is measured over time (i.e., up to six months or one year), and the primary outcomes are the curve (i.e., expansion versus time) and the plateau of the expansion process [64].

Two main testing procedures can be used to measure the residual expansion of ISR-affected concrete. The first setup involves storing the samples at 95% relative humidity and 38 °C. A second setup specifies that the samples be maintained in a 1 M NaOH solution at 38 °C; this method is recommended for determining the residual aggregate reactivity. Compared to the second method, the first method is more reliable in determining the residual concrete expansivity since it does not add more alkalis to the system than the concrete itself. Therefore, the concrete reaches its own capacity to expand. The second method, however, improves the swelling potential of the aggregate by adding more alkalis into the system [64]. Despite these methods’ valuable outcomesthey still present some challenges for interpretation; for example, there is no correlation data between laboratory and field results, a lack of correlation between laboratory temperatures and field temperatures, a higher humidity level in the laboratory than in the field, and cores with defects such as pores that do not represent the real structure.

#### 6.2.2. Soluble Alkalis

The residual alkali content can be used to forecast whether ISR-affected concrete will expand further or not since the availability of alkalis is related to ASR development. In other words, as seen in Table 3, the pH of the pore solution plays a crucial role in ISR kinetics, both to initiate new reactions and maintain ongoing reactions. The authors in [65] compared five methods to determine the pH and the free alkali content: pore water expression (PWE), in situ leaching (ISL), ex situ leaching methods (ESL), cold water extraction (CWE), hot water extraction (HWE), and espresso. There is a consensus among the authors that the espresso method provides the most promising results. However, sampling is one of the most important points in determining the alkali content. In this sense, the alkali content should be extracted from members at different depths of the structure under analysis because it may show expressive differences.

## 7. Modeling

The mathematical models developed to predict the behavior of concrete structures distressed by ISR were classified into four groups: micromodels (based on ion diffusion/reaction products), micro-mesomodels (based on gel production), mesomodels (based on internal pressure), and macro-models (based on concrete expansion) [66], where the first three are considered on the material scale and the fourth one on the structural scale.

Additionally, the most cited mathematical model in the materials scale was the one proposed by [67]. A representative elemental volume of concrete (REV) is used as the basis for this model, considering some chemical mechanisms, such as (a) the effects of alkali diffusion into aggregate particles, (b) an increase in ASR gel production with an increase in the concentration of alkali in aggregate particles, (c) ASR gel consumes alkali from the cement paste, decreasing the cement paste alkali concentration, and (d) ASR gel is displaced into the porous zone surrounding the affected aggregate particles in bulk cement paste. Whenever ASR gel produces stresses greater than concrete’s tensile strength, cracks and damage occur in the cement paste surrounding the affected aggregate particles.

Recently, Nguyen et al. [68] developed a model prior to that proposed by de Grazia et al. [69], which implemented measurable parameters, such as temperature, aggregate reactivity, humidity, and alkali content, into Larive’s model [70]. Nguyen et al. [68] implemented alkali leaching into the model and evaluated the short-term expansion of laboratory specimens versus the long-term expansion of field blocks, finding solid correlations.

Furthermore, several macro-models (i.e., structural scales) have been proposed in the past decades. Some of them are considered neither overcomplicating nor oversimplifying the physicochemical aspects of the reaction, such as Gorga [71] and Gorga et al. [4] and Gorga et al. [72], which describe an engineering-based finite element approach to appraise ASR-affected structures and validate the estimation of slender structural members and the expansion behavior of a massive dam, respectively.

## 8. Discussion

The bibliometric analysis reveals that the diagnosis and prognosis of ISR-affected structures rely on a variety of field and testing methodologies. While each technique may provide valuable outputs for maintenance and forecasting the structures’ behavior, an efficient and accurate protocol is required for assertive management.

### 8.1. Enhancing Diagnosis and Prognosis of ISR-Affected Structures

Traditional visual inspections, while fundamental to initial assessments, offer a limited qualitative overview of concrete structures’ surface conditions. Their effectiveness is complemented by quantitative techniques, such as the cracking index (CI), which offers measures of external damage. While useful, especially when employed over time, it fails to indicate the internal condition of concrete or identify the potential deterioration mechanism. This is evident when evaluating the variable correlation between surface cracking expansion and internal expansion in structures subjected to the same deterioration mechanism (ASR) and exposed to the same real environmental conditions [56].

The limitations of surface-level observations require the incorporation of non-destructive techniques (NDTs), offering insights into the integrity of ISR-affected structures. Since concrete properties have been extensively correlated with sound propagation and electrical resistivity, NDTs can serve as a comparative indicator between sound and damaged areas. In this sense, the results of NDTs are limited to the concrete integrity and are not able to indicate the actual cause of the damage.

Nonetheless, a multi-level assessment approach that includes microscopic and mechanical tests provides a thorough understanding of both the cause and extent (i.e., diagnosis) of a deteriorated concrete structure. For instance, stiffness damage testing (SDT) and damage rating index (DRI) have established a relationship between the degree of damage and the resulting damage, as summarized in Table 4 [22]. Such approaches have proved efficient in assessing real structures in a range of aged infrastructures, including 50 to 85-year-old dams, 90-year-old river walls, and 65-year-old bridges affected by AAR, offering a direct correlation between expansion levels and the outputs of DRI and SDT [56,73,74].

In this sense, diagnosing ISR-affected structures would benefit from DRI and SDT’s ability to correlate testing results with sample conditions (i.e., damage degree). These outcomes relate directly to reductions in stiffness, compressive strength, and tensile strength, offering a reference to the expansion level (%). Additionally, as STD operates at 40% of the 28-day concrete compressive strength, it allows for the reuse of limited samples in further tests like residual expansion and petrographic analysis [57].

As the diagnosis has been established, the current challenge of an efficient management protocol for ISR-affected structures demands a comprehensive prognosis procedure integrating laboratory test results. The potential of using residual expansion and soluble alkalis test outcomes to predict future behavior is based on the assessment of AAR-affected dams [75]. Yet, it is still to be explored in a range of field conditions to be validated for other ISR mechanisms.

In such a scenario, combining laboratory outcomes with modeling from the materials perspective may indicate the remaining service life of the structures. Moreover, monitoring techniques coupled with assertive diagnosis and prognosis are needed to plan the appropriate timeframe for maintenance and interventions on ISR-affected infrastructure. In summary, current protocols, limited to petrographic examination, superficial crack evaluation, and chemical analysis, would benefit from incorporating DRI, SDT, field monitoring, residual expansion or soluble alkalis, and modeling.

### 8.2. Proposed Flowchart to Guide the Diagnosis and Prognosis of ISR-Affected Critical Infrastructure

**Figure 7:**
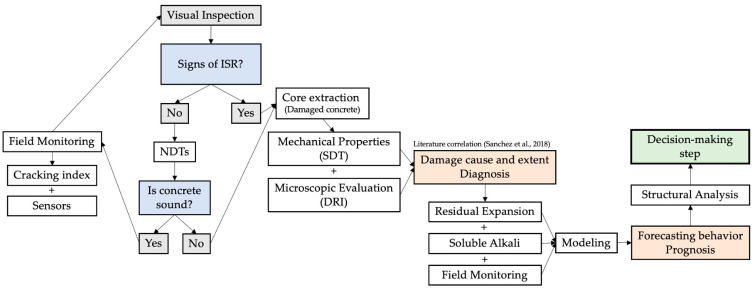
Proposed flowchart to guide the diagnosis and prognosis of ISR-affected critical infrastructure.

Based on the discussion raised, Figure 7 presents a structured guide for the diagnosis and prognosis of ISR-affected critical infrastructure. In fact, the proposed flowchart serves as an initial step toward the efficient management of ISR-affected structures. The approach implements routine visual inspections and field monitoring as fundamental practices, aiming to early detect any deterioration mechanism.

If indicators of ISR are detected and integrity testing is compromised, core samples must be taken for further diagnosis. Once the cause and extent are known, residual expansion, soluble alkalis, and field monitoring are to be explored. Such inputs serve for the modeling process, aiming to indicate the remaining service life of the structure, as well as maintenance requirements. Finally, structural analysis may be required and considered to ensure the structure’s safety and the need for rehabilitation strategies.

This framework, while progressing towards a comprehensive protocol, acknowledges the need for further refinement. The topic would also benefit from advanced predictive methodologies, such as machine learning and stochastic modeling, to delineate thresholds and the efficacy of the proposed measures across varying ISR mechanisms and structures.

## 9. Conclusions

This paper aims to present a comprehensive literature review of the methodologies for assessing and predicting ISR-affected infrastructures. In summary, the work reached the following conclusions:

- The primary step in assessing ISR-damaged concrete structures (fields) focuses on visual inspection, along with cracking measurement and non-destructive techniques. In laboratory analysis, microscopic techniques such as petrography, epoxy fluorescent-coated sections, DRI, QDA, and mechanical tests such as SDT and tensile tests are the main ones.
- Recent advancements have emphasized multi-level assessment techniques by combining SDT and DRI for a comprehensive understanding of the damage cause and extent (i.e., diagnosis).
- The prognosis of ISR-damaged concrete relies on monitoring field strategies aligned with laboratory techniques, such as residual expansion and soluble alkalis. In which both field and laboratory outcomes are input into modeling to forecast the future behavior of ISR-affected structures.
- It is suggested that an effective protocol for accessing ISR-affected critical infrastructure should combine robust diagnosis and prognosis techniques addressed in this research, such as DRI, SDT, field monitoring, residual expansion, soluble alkalis, and modeling predictions.
- The flowchart proposed in this study may serve as a guide for diagnosis and prognosis for ISR-affected structures. Yet probabilistic approaches may establish limits and refine the protocol.

In summary, this review underscores the shift towards integrated and advanced methodologies for accurately assessing and predicting the impact of ISR on infrastructure.

## Figures and Tables

**Table 1:** Petrographic features and corresponding weighting factors for DRI [19].

Petrographic Features		Weighting Factors
Closed crack in aggregate	CCA	0.25
Opened crack in aggregate	OCA	2
Crack with reaction product in coarse aggregate	OCAG	2
Coarse aggregate debonded	CAD	3
Disaggregate/corroded aggregate particle	DAP	2
Crack in cement paste	CCP	3
Crack with reaction product in cement paste	CCPG	3

**Table 2:** Summary of diagnosis methods for ISR damage.

Method	Outcomes	Sample Specifications	Reference
Petrography (Thin section)	Concrete’s composition, texture, microstructure, and physicochemical properties	Thin section, 20 and 30 μm thickness, 75 × 40 mm^2^	[51]
Epoxy Fluorescence	Cracks or other signs of distress captured with high precision	Thin section, 20 and 30 μm thickness, 75 × 40 mm^2^	[51]
DRI	DRI number, which, in combination with mechanical properties losses, can be used to identify the damage caused	200 cm^2^, counted in 1 cm^2^ grids	[19]
QDA	Damage degree classification from 1 to 5	5 thin sections	[54]
SDT	Damage extent, mechanical properties losses, physical integrity, and cracking orientation, indicated by ME, H, NLI, SDI, PDI	Length to diameter should be equal to two.	[55,56]
Tensile	Tensile strength, microstructure net cracking, and physical integrity	Cylindrical specimens.	[58,60]

**Table 3:** Summary of prognosis methods for ISR damaged structures.

Method	Outcomes	Sample Specifications	Reference/Standard
Residual expansion	Residual expansion curve behavior and the plateau of remaining ISR expansion	Core extracted from the structure under investigation	[64]
Soluble alkalis	Residual alkalis content and the pore solution pH	Homogeneous ground concrete from the structure under investigation	[65]

**Table 4:** Classification of the damage degree in concrete due to ASR, FT, and DEF [22].

Distress Mechanism	Classification of ASR Damage Degree (%)	Reference Expansion Level (%)	Damage Results				
			Stiffness Loss (%)	Compressive Strength Loss (%)	Tensile Strength Loss (%)	SDI	DRI
ASR	Negligible	0.00–0.03	–	–	–	0.06–0.16	100–155
	Marginal	0.04 ± 0.01	5–37	(−)10–15	15–60	0.11–0.25	210–400
	Moderate	0.11 ± 0.01	20–50	0–20	40–65	0.15–0.31	330–500
	High	0.20 ± 0.01	35–60	13–25	45–80	0.19–0.32	500–765
	Very high	0.30 to 0.50 ± 0.01	40–67	20–35		0.22–0.36	600–925
	Ultra-high	0.50 to 1.00 ± 0.01	–	–	–	–	–
		≥1.00 ± 0.01	–	–	–	–	–
FT and FT + ASR	Negligible	0.00–0.03	–	–	–	11	147–154
	Marginal	0.04 ± 0.01	23–35	12–32	44–67	16–23	496–684
	Moderate	0.11 ± 0.01	28–36	21–32	62–67	25–28	590–950
	High	0.20 ± 0.01	33–46	22–37	65–67	27–41	677–963
	Very high	0.30 to 0.50 ± 0.01	37–52	24–40	65–73	34–45	800–1300
	Ultra-high	0.50 to 1.00 ± 0.01	–	–	–	–	–
		≥1.00 ± 0.01	–	–	–	–	–
DEF and DEF + ASR	Negligible	0.00–0.03	–	–	–	11	110–147
	Marginal	0.04 ± 0.01	–	–	–	–	–
	Moderate	0.11 ± 0.01	35–56	9–34	–	17–20	355–599
	High	0.20 ± 0.01	–	–	–	–	–
	Very high	0.30 to 0.50 ± 0.01	55–62	29–43	–	19–28	623–710
	Ultra-high	0.50 to 1.00 ± 0.01	56–77	40–47	–	27–43	828–1022
		≥1.00 ± 0.01	60–86	40–50	–	30–54	841–1363

## Data Availability

Data are contained within the article.
